# Phage Therapy for *Mycobacteria*: Overcoming Challenges, Unleashing Potential

**DOI:** 10.3390/idr17020024

**Published:** 2025-03-12

**Authors:** Christoffel Johannes Opperman, Adrian J. Brink

**Affiliations:** 1 National Health Laboratory Service, Green Point TB-Laboratory, Cape Town 8005, South Africa; stefan.opperman@uct.ac.za; 2 SAMRC Centre for Tuberculosis Research, Division of Molecular Biology and Human Genetics, Stellenbosch University, Cape Town 7505, South Africa; 3 Division of Medical Microbiology, Department of Pathology, University of Cape Town, Cape Town 7925, South Africa; 4 Institute of Infectious Disease & Molecular Medicine, University of Cape Town, Cape Town 7925, South Africa

## 1. Introduction

Bacteriophage (phage) therapy is emerging as a promising alternative to traditional antibiotics for treating drug-resistant mycobacterial infections, including *Mycobacterium tuberculosis* complex (MTBC) and non-tuberculous mycobacteria (NTM) [1]. Phages offer key advantages such as high bacterial strain specificity, reducing harm to the host microbiota, biofilm penetration, rapid bactericidal action, and cost-effective large-scale production [2]. These attributes make phage therapy a potential solution in combating drug-resistant mycobacterial infections.

## 2. Overcoming Challenges

Despite its potential, several challenges hinder the clinical application of mycobacteriophage therapy. A key limitation is the narrow host range of many mycobacteriophages, restricting their ability to target diverse and genetically variable *Mycobacterium* strains [3]. Additionally, limited knowledge of bacterial receptors essential for phage attachment further complicates treatment efforts. Advances in sequencing and bioinformatics could help identify new receptor-binding sites, broadening the spectrum of effective phages [4]. Phage cocktails, which combine multiple phages targeting different bacterial receptors, offer a promising strategy to expand therapeutic coverage and mitigate resistance development [5]. Additionally, combining phage therapy with antibiotics may enhance treatment efficacy by slowing resistance emergence and reducing bacterial adaptation costs [1].

To mitigate phage resistance, personalized treatment regimens should be prioritized over generalized approaches [6]. An innovative strategy involves using “enzybiotics”, such as chimeolysins and artilysins, which are bioengineered enzymes designed to enhance antibacterial activity beyond whole phages by targeting bacterial cell walls more effectively [7]. Additionally, phage-based components, such as nanoparticle-delivered endolysins and engineered enzymes capable of crossing epithelial membranes, present promising solutions to improve therapeutic efficacy [8]. These advancements not only enhance treatment potential but also create opportunities for pharmaceutical investment in phage-based therapies.

The lack of standardized regulatory frameworks hinders the clinical adoption of phage therapy [9]. In this regard, the European Committee on Antimicrobial Susceptibility Testing (EUCAST) is reviewing the current progress in phage therapy and establishing whether a reliable susceptibility testing method can be developed. Unlike antibiotics, phage therapy lacks universal approval guidelines, making its implementation challenging. Developing flexible, globally harmonized regulations is essential to facilitate clinical use, particularly for compassionate cases where no other treatments are available. Additionally, establishing global phage biobanks and databases with well-characterized and sequenced phages would expedite the selection process, enhancing therapeutic accessibility and efficiency [10].

The challenge of targeting intracellular mycobacteria within macrophages limits the effectiveness of phage therapy [11]. Mycobacteria can survive and replicate inside macrophages, making them less accessible to phages [12]. To address this, strategies such as engineering polyvalent phages with receptor-binding proteins, using hybrid enzybiotics like innolysins, and employing liposome encapsulation to enhance phage delivery have been proposed [13,14]. Additionally, the “Trojan horse” strategy, utilizing non-virulent bacterial strains like *Mycobacterium smegmatis* as phage delivery vectors, presents an innovative approach to overcoming intracellular barriers [12].

In vitro and clinical studies also face significant challenges due to the limitations of current experimental models, which often fail to replicate the complex pathophysiological conditions of mycobacterial infections. Mycobacteria thrive in environments characterized by low oxygen levels and nutrient deprivation, such as granulomas, which are difficult to simulate in standard laboratory settings [15]. To improve the prediction of phage efficacy in vivo, it is essential to develop experimental models that better mimic these disease environments. Such models would provide more reliable data on the performance of phage therapy under conditions that more closely resemble those encountered in actual infections, thereby improving the translation of preclinical findings to clinical practice.

Immune neutralization, where the host immune system produces antibodies that inactivate phages, has the potential to reduce the therapeutic effectiveness of mycobacteriophages [16]. This can be particularly problematic when phages are administered over extended periods. Potential solutions include characterizing phage cocktails for potential cross-reactivity before clinical use, ensuring that each phage in the mixture remains effective [17]. Intermittent or serial dosing regimens could also be employed to prevent the simultaneous neutralization of all phages, thereby preserving therapeutic efficacy [18]. Furthermore, genetically engineered phages designed to enhance lytic activity, along with the use of natural phage mutants that have undergone selection for broader host infectivity, may reduce the impact of immune neutralization [1]. In addition, the complex interactions between mycobacteria and the lung environment also warrant further investigation. Alveolar lining fluid, which contains enzymes and surfactants, can alter the structure of the mycobacterial cell envelope, potentially affecting the ability of phages to bind to and infect *M. tuberculosis* [19]. Understanding these interactions, particularly in the context of comorbidities such as Human Immunodeficiency Virus (HIV) or diabetes mellitus [20], is essential for optimizing phage therapy in diverse patient populations.

## 3. Conclusions

Mycobacteriophage therapy shows great promise as an alternative or adjunct to antibiotics for drug-resistant mycobacterial infections. However, challenges such as phage specificity, resistance development, regulatory hurdles, intracellular access, immune neutralization, and the absence of standardized clinical models remain. Advances in bioengineering, regulatory reform, and mycobacterial pathophysiology research offer pathways to overcome these barriers. By addressing these challenges, phage therapy could revolutionize the treatment of MTBC and NTM infections. Continued research, collaboration, and innovation will be key to unlocking its full therapeutic potential.
